# Developing a SNOMED CT–Based Value Set to Document Symptoms and Diagnoses for Adverse Drug Events: Mixed Methods Study

**DOI:** 10.2196/70167

**Published:** 2025-07-08

**Authors:** Erica Y Lau, Linda Bird, Anthony Lau, Yau-Lam Alex Chau, Katherine Butcher, Susan Buchkowsky, Kira Gossack-Keenan, Cheryl Sadowski, Corinne M Hohl

**Affiliations:** 1 Strategy, Planning and Implementation, BC Cancer, 601 West Broadway, Vancouver, BC, V5Z 4C2, Canada, 1 6048776000; 2 Department of Health Information Science, University of Victoria, Victoria, BC, Canada; 3 Pharmaceutical Science, Vancouver General Hospital, Vancouver, BC, Canada; 4 Faculty of Medicine and Dentistry, University of Alberta, Edmonton, AB, Canada; 5 Pharmacy Department, Surrey Memorial Hospital, Surrey, BC, Canada; 6 Vancouver General Hospital, Vancouver, BC, Canada; 7 Faculty of Pharmacy & Pharmaceutical Sciences, University of Alberta, Edmonton, AB, Canada; 8 Department of Emergency Medicine, The University of British Columbia, Vancouver, BC, Canada; 9 Vancouver Coastal Health Research Institute, Centre for Clinical Epidemiology and Evaluation, Vancouver, BC, Canada

**Keywords:** adverse drug event, electronic medical records, pharmacovigilance, terminology standard, SNOMED CT

## Abstract

**Background:**

Adverse drug events (ADEs) lead to more than 2 million emergency department visits in Canada annually, resulting in significant patient harm and more than CAD $1 billion in health care costs (in 2018, the average exchange rate for 1 CAD was 0.7711 USD; 1 billion CAD would have been approximately 771.1 million USD). Effective documentation and sharing of ADE information through electronic medical records (EMRs) are essential to inform subsequent care and improve safety when culprit medications can be replaced and reexposures avoided. Yet, current systems often lack standardized comprehensive ADE value sets.

**Objective:**

This study aimed to develop a SNOMED CT value set for symptoms and diagnoses to standardize ADE documentation and improve ADE data integration into EMRs.

**Methods:**

We used ADE data from ActionADE, a prospective reporting system implemented in 9 hospitals in British Columbia. We extract 5792 reports that yielded 827 unique ADE symptom and diagnosis terms based on Medical Dictionary for Regulatory Activities preferred terms. Two independent mappers used both automated and manual mapping approaches to match these terms to SNOMED CT concepts. Two clinical experts conducted validation, followed by a quality assurance review by a separate clinical team. Discrepancies were resolved through consensus discussions. Interrater reliability was assessed using Cohen κ.

**Results:**

The automated mapping process identified 63.1% (522/827) semantically equivalent matches from SNOMED CT’s Clinical Finding hierarchy. Two mappers manually reviewed the automatically mapped terms and identified appropriate target concepts for the unmapped terms. After the manual mapping process, 95.3% (788/827) of the source terms were successfully mapped to SNOMED CT concepts, with 4.7% (39/827) remaining unmapped. Interrater reliability between the mappers was strong (κ=0.87, 95% CI 0.85‐0.89). The validation phase identified and removed 1 irrelevant term, resulting in 98.4% (813/826) terms mapped, with 1.6% (13/826) unmapped, and a high interrater reliability (κ=0.88, 95% CI 0.80‐0.95). During quality assurance, 6 terms were flagged for concerns regarding clinical relevance or safety risks and were resolved through discussions. The final value set comprised 813 SNOMED CT concepts, with 95.7% (778/813) of terms classified as semantically equivalent and 4.3% (35/813) as semantically similar. Thirteen additional terms remained unmapped and will be reviewed as new SNOMED CT codes are added.

**Conclusions:**

This study developed a SNOMED CT–based value set to document symptoms and diagnoses for ADEs observed in adults in EMRs. Adopting this value set can improve the consistency, accuracy, and interoperability of ADE documentation in EMRs, helping to reduce repeat ADEs and enhance patient safety. Ongoing refinement and improved clinical usability are essential for its widespread adoption. Future research should assess the impact of integrating this value set into EMRs on ADE reporting, pharmacovigilance, and patient safety outcomes.

## Introduction

Every year, more than 2 million Canadians visit an emergency department due to adverse drug events (ADEs)—unintended and harmful events related to medication use [1,2]. These events lead to more than 700,000 hospital admissions and exceed CAD $1 billion in health care costs across Canada annually (in 2018, the average exchange rate for 1 CAD was 0.7711 USD. 1 billion CAD would have been approximately 771.1 million USD) [2,3]. Critically, 75% of these events are preventable [4,5]. A common cause of preventable ADEs is the represcription or redispensing of the same or a similar class of medication that previously caused harm, which may or may not be warranted [6,7]. Addressing unintentional repeat ADEs is essential to enhancing patient safety and aligns with the World Health Organization’s public health priorities [8].

Unintentional repeat ADEs may occur because health systems lack effective mechanisms to communicate and integrate ADE information into clinical workflows [9]. For example, community pharmacists lack access to hospitals’ electronic medical records (EMRs) and do not receive discharge summaries or consultation notes, leaving them unaware of ADEs diagnosed in hospital where safer alternatives may exist and where reexposure should be avoided [10,11]. This communication gap presents a critical opportunity to reduce repeat ADEs through improved data-sharing practices [12].

The widespread adoption of EMRs offers an opportunity to enhance the documentation and sharing of ADE information among health care providers [13-15]. However, for this information to be effectively shared across clinical disciplines and care settings and follow the patients along their care trajectory, it must be recorded, stored, and shared in a standardized form. Currently, most EMRs include allergy modules for documenting symptoms and diagnoses related to ADEs. Allergies constitute a small percentage of clinically significant ADEs, and many are subsequently refuted [16]. Current EMR modules often rely on nonstandardized adverse reaction code sets provided by third-party vendors, with varying quantity, quality, and data standards. This hinders the uniform documentation, extraction, and sharing of ADE data [17]. Inconsistent and nonstandardized ADE documentation can lead to underreporting and miscommunication, contributing to adverse patient outcomes and posing challenges for population-level pharmacovigilance efforts. Studies have shown that ADEs are responsible for 6%-27% of adult patients’ hospital or acute medical unit admissions, with a substantial portion being preventable through accurate tracking and effective communication [18,19].

Adopting standardized value sets can bridge these discrepancies and improve the reliability of clinical documentation [17]. This standardization is particularly important in reducing unwarranted clinical variation, ultimately leading to more consistent and evidence-based care [20]. A value set is a collection of coded values from 1 or more terminologies that is intended for use in a particular context [21]. Selecting terminologies with broad applicability across a range of clinical areas can help create robust and effective value sets.

While several terminology standards are available to document symptoms and diagnoses of ADEs, not all are ideal for use in EMRs due to limitations in granularity, hierarchical design, and semantic expressivity. For example, the Medical Dictionary for Regulatory Activities (MedDRA) and the *International Classification of Diseases, Tenth Revision* (*ICD-10*) have limitations that impede effective data capture and integration within EMRs. Although MedDRA is designed for pharmacovigilance, its terms are categorized at high level without distinguishing specific conditions, limiting its clinical use. Its hierarchical structure is broad and fixed, limiting its flexibility to support detailed data retrieval and analysis [22,23]. Bodenreider [24] has discussed its limitations when used in isolation and the potential benefits of combining it with SNOMED CT for improved signal detection and ADE reporting. Similarly, *ICD-10* is primarily designed for billing and statistical reporting. Its broad disease categories can fail to capture the nuances of complex clinical relationships, such as disease severity, laterality, or specific symptoms, lacking the clinical granularity needed for detailed ADE documentation within patient records. These limitations impact real-time decision-making [25-27].

In contrast, SNOMED CT (Clinical Terms) is specifically developed to support detailed clinical documentation and interoperability across various health care settings. It offers more than 300,000 active concepts that cover a broad range of clinical areas, allowing for precise and consistent recording of patient information [28-30]. SNOMED CT key strengths lie in its granularity, polyhierarchy, and semantic expressivity. making it a preferred choice as the basis of clinical documentation within EMRs across multiple jurisdictions [31]. Alecu et al [32] demonstrated that SNOMED CT’s hierarchical structure is particularly useful for grouping ADE terms, which can aid in clinical decision-making and data analysis. However, if left unconstrained, its vast size and complexity can be overwhelming for end users. Therefore, SNOMED International recommends the development of subsets or value sets that cater to specific clinical use cases, such as ADEs.

There are currently no internationally published SNOMED CT value sets designed to streamline and standardize clinical documentation of ADE symptoms and diagnoses. This study aimed to fill this gap by developing a SNOMED CT–based value set to document and code symptoms and diagnoses of ADEs in EMRs. By doing so, we can enhance the reliability and consistency of ADE documentation and improve patient safety.

## Methods

### Setting

We used ADE data from ActionADE, an ADE reporting system. ActionADE is a web-based application designed to address repeat ADEs, which occur in 32.5% (421/1296) of emergency department cases. Repeat ADEs often occur because health systems lack effective means of communicating and integrating ADE information into clinical workflows [10]. A systematic review, participatory design process and pilot testing, involving software developers and end users, was completed to optimize the system to integrate ADE information into clinical workflows [11,33]. ActionADE allows providers to document and communicate ADE information bidirectionally through its integration with the provincial drug-dispensing database (PharmaNet) to providers in other health settings. Preliminary results showed that ActionADE prevents culprit or same-class medication redispensations in 33% of patients who present to a community pharmacy with a culprit or same class medication prescription [34].

ActionADE contains patient’s ADE information entered by clinicians across acute care hospitals serving adult populations within the Vancouver Coastal Health Authority, British Columbia, since 2021 [25]. Between May 1, 2021, and May 1, 2024, ActionADE captured 5900 ADE reports. After excluding 108 reports with missing symptom and diagnosis information, we extracted source terms from a final sample of 5792 reports.

### Data Extract and Processing

Each of the 5792 reports contained up to 3 ADE symptoms and diagnoses, which we extracted into an Excel file. After removing duplicates, we identified 881 unique terms. ActionADE uses MedDRA preferred terms for documenting ADE symptoms and diagnoses, based on its usability, and as recommended for reporting to Health Canada’s pharmacovigilance database [35]. Since data entry into ActionADE is restricted to the use of MedDRA preferred terms, data are already standardized and free from potential data entry errors. We linked the 881 terms to MedDRA numeric codes, resulting in 879 coded terms and 2 uncoded terms. Upon review, we removed the 2 uncoded terms as they were noncurrent. Additional adjustments, such as removing irrelevant terms and duplicate entries, updating display names, and merging terms with different spelling but the same codes, resulted in a final source code set of 827 terms.

### Mapping Approach

Our mapping approach was informed by the SNOMED CT-AU mapping guide [36] and enhanced by additional resources [36-38]. It consisted of the following steps: (1) define purpose and scope of the map, (2) identify personnel, (3) define mapping rules, (4) select mapping tool, (5) map source to target (automated and manual), (6) validation, and (7) quality assurance and creation of the value set.

### Define Purpose and Scope of the Map

Defining the purpose and scope of the map helps ensure that the mapping aligns with the intended use of the terminology and covers the necessary range of source and target codes with the appropriate granularity [30,36]. The purpose of this mapping is to standardize the documentation of symptoms and diagnoses of ADEs within EMR. This involves mapping ADE symptoms and diagnoses documented in MedDRA preferred terms to corresponding SNOMED CT concepts within the clinical finding hierarchy.

### Identify Personnel

The mapping team should be composed of individuals with extensive knowledge in health terminology standards and clinical practice to ensure the effective management and accuracy of the mapping process. In this study, the mapping process was designed and overseen by a mapping manager with specialized graduate training in health terminology standards. Execution was led by 2 mapping team members (Y-L AC and AL), both research pharmacists with extensive experience in clinical applications and practice. The team also included 2 clinical map advisors (CH and KB): an emergency physician and health services researcher specializing in drug safety and health IT, and a clinical pharmacist with extensive research and clinical practice experience in this area. Additionally, a technical advisor (LB), who is an expert in health informatics and SNOMED CT, provided essential technical oversight and support.

### Define Mapping Rules

This unidirectional mapping from MedDRA to SNOMED CT involved matching each MedDRA preferred term to a semantically equivalent SNOMED CT concept (ie, they mean the same thing), ensuring accuracy in the representation of clinical data.

### Select Mapping Tool

We used Snap2SNOMED, a mapping tool provided by SNOMED International, to conduct the mapping [39]. Snap2SNOMED is an online platform designed for creating and maintaining simple maps from other code systems to SNOMED CT. It allows teams of users to author and review a map. It has a built-in search engine that allows users to browse SNOMED CT concepts while mapping. Users can import their own code sets and export maps in various formats, such as CSV, TSV, and XLSX. Snap2SNOMED allowed us to import our source term file, use the MedDRA-SNOMED CT cross map, and automate much of the mapping process, which was then reviewed and refined by the team.

### Map Source to Target

We used a dual mapping process where 2 mappers independently identified and documented semantically equivalent SNOMED CT concepts for each source term. If an exact match was unavailable, the mappers documented up to 3 semantically similar concepts, marking them as inexact, broader, or narrower matches. For example, “heart attack” is a semantically equivalent match to “22298006 | Myocardial infarction” as both represent the exact same clinical condition. A narrower match occurs when “heart attack” is mapped to “57054005 | Acute myocardial infarction (disorder),” which represents a specific type of myocardial infarction. When selecting a target code, the mappers reviewed the concept’s fully specified name (FSN) and its parent and child concepts within the hierarchy to ensure semantic accuracy. The mapping process involved two key strategies:

1. Automated mapping: We first used the MedDRA-SNOMED CT cross map to identify appropriate target concepts. This cross map was developed by SNOMED International in collaboration with the International Council for Harmonisation of Technical Requirements for Pharmaceuticals for Human Use. This map supports interoperability between terminologies by linking commonly used pharmacovigilance terms from MedDRA to SNOMED CT. The cross map released on April 30, 2024, includes 6779 MedDRA lower-level terms and 3594 SNOMED CT FSNs [5]. While ActionADE relies on MedDRA preferred terms, each preferred term is associated with a single lower-level term, so this did not impact our mapping process [22].
  The mapping manager imported the source code set and the cross map into Snap2SNOMED. The map was constrained to the Canadian Edition (version: 20240531) and the SNOMED CT clinical finding hierarchy. The software automatically mapped the source terms to SNOMED CT concepts within the cross map. Then, it generated a table with each row representing a single source term, displaying its corresponding source code, source display, target code, and target display. For matched terms, the relationship type was default as “EQUIVALENT,” with the status marked as “DRAFT.”
2. Manual mapping: For each automatically mapped term, the mappers independently reviewed and edited the suggested target terms and relationship types, updating the status to “MAPPED” upon completion.
  For source terms that were unmatched in the automated mapping, the mappers used Snap2SNOMED’s built-in search function to identify target terms. By entering the source term in the search box, the software provided a list of suggested target concepts. The mappers reviewed the suggested terms and their attributes, selected the most appropriate candidate concept, assigned it to the source code with a relationship type (equivalent, broader, narrower, and inexact), and marked the status as “MAPPED” or “NO MAP” if no suitable match was found. For the source terms that lacked semantically equivalent matches, the mappers were asked to suggest up to 3 broader, narrower, or inexact alternatives (see Snap2SNOMED screenshots in Multimedia Appendix 1).

The mapping manager then exported and compared the maps from both mappers. A mapping was considered complete when both mappers independently identified the same SNOMED CT concept as the target. Any discrepancies were discussed and resolved through a virtual meeting moderated by the mapper manager. If consensus could not be reached, the issues were documented and adjudicated by the clinical advisors [36].

### Validation

Two validators (CH and KB) independently evaluated the initial map using Snap2SNOMED. For each row, they reviewed the mapped terms and marked the status as either “ACCEPTED” or “REJECTED,” recording the rationale for any rejections in the notes section. A map was considered correct only when both reviewers agreed on the target SNOMED CT concept (Multimedia Appendix 1). The mapping manager then exported and compared the results from both validators, consolidating a list of discrepancies. A virtual meeting was held to discuss and resolve these discrepancies. If consensus could not be reached, the issues were escalated to an additional reviewer and, if necessary, further to the technical advisor.

### Quality Assurance and Creation of the Value Set

Quality assurance was performed by clinical experts who were not involved in the initial mapping and validation process [30,38]. A quality review team, consisting of 2 pharmacists (SB and CS) and a physician (KG-K), evaluated the final map for mapping appropriateness, safety risk, and clinical relevance. Each reviewer reviewed all the terms included in the validated map and flag terms with concerns by adding a note on the Snap2SNOMED platform.

As a final step, the reviewers completed a survey to assess clinical relevance of the validated map. The survey included two questions: (1) Does the current map capture the majority of ADE symptoms and diagnoses commonly encounter in your clinical practice? If not, which key terms are missing? (2) Are there any terms in the map that seem clinically irrelevant to your practice, or do you have suggestions for alternative terms that should be considered in future iterations? Terms flagged by 2 or more reviewers were discussed by the entire team to reach a consensus. This feedback was then used to finalize the value set.

### Ethical Considerations

This study is exempt from ethical review according to The University of British Columbia Research Ethics Board policies (sections 4.3.2. and 4.3.3) [40] and TCPS 2 Article 2.3 [41]. The ActionADE dataset used for developing the MedDRA-SNOMED CT map was collected under prior ethical approval, with informed consent obtained from participants allowing for secondary research use. All data were deidentified. Individuals involved in the mapping and validation participated voluntarily, were informed of the study purpose, and provided implied consent. No personal identifiers were collected or shared, no compensation was provided, and the activities posed minimal risk; therefore, ethics review was not required.

### Statistical Analysis

We used descriptive statistics to calculate the number and percentage of source terms that matched a corresponding SNOMED CT concept by relationship type in the initial map and validated map. The agreement between mappers was evaluated by checking whether their first selected SNOMED CT concept for each source term matched (first match). Interrater reliability across 2 mapping reviewers and 2 validators was calculated using Cohen κ statistics via the R package “irr” (University of Zurich) [42].

## Results

### Map Source Terms to SNOMED CT Concepts

The automated mapping identified 63.1% (522/827) semantically equivalent matches and 36.9% (305/827) unmatched terms. After manual mapping, mapper 1 identified 91.1% (753/827) semantically equivalent matches, 5.7% (47/827) semantically similar matches, and 3.2% (27/827) no match terms. Mapper 2 identified 85.0% (703/827) semantically equivalent matches, 8.0% (66/827) semantically similar matches, and 7.0% (58/827) no match terms (Table 1). All automatically mapped terms were accepted by both mappers.

For the source terms that lacked semantically equivalent matches, the mappers were asked to suggest up to 3 broader, narrower, or inexact alternatives. Mapper 1 suggested 57 target terms for 47 source terms without equivalent matches, averaging 1.2 suggestions per term. Mapper 2 proposed 66 target terms for 66 source terms, averaging 1.0 suggestion per term. A detailed breakdown of the semantically similar terms suggested by the mappers is shown in Table 2. The interrater reliability between the 2 mappers, measured by Cohen κ, was 0.87 (95% CI 0.85‐0.89), indicating strong agreement [43].

We identified 139 terms with discrepancies between the mappers. These were all resolved through consensus discussions. Based on these results, we created an initial map that included 827 source terms, of which 95.3% (788/827) were successfully mapped to a SNOMED CT concept, while 4.7% (39/827) remained unmapped. Among the mapped terms, 96.8% (763/788) were semantically equivalent, 1.5% (12/788) were broader, 0.8% (6/788) were narrower, and 0.9% (7/788) were inexact matches.

**Table 1.** Mapping results across all source terms.

Category	Mapper 1	Mapper 2
Semantic equivalent match	753	703
Semantic similar match	47	66
No match	27	58
Total	827	827

**Table 2.** Breakdown of semantically similar terms suggested by mappers.

Category	Mapper 1	Mapper 2
Semantically broader than the source term	23	12
Semantically narrower than the source term	24	25
Semantically inexact to the source term	10	29
Total	57^a^	66^b^

a Mapper 1 suggested 57 target terms to describe 47 source terms without a semantically equivalent match, resulting in an average of 1.2 target terms per source term.

b Mapper 2 suggested 66 target terms to describe 66 source terms without a semantically equivalent match, resulting in an average of 1.00 target terms per source term.

### Validation

Validation by 2 clinical experts resulted in 92.3% (763/827) of terms being accepted as semantically equivalent matches, 3.0% (25/827) were semantically similar, and 4.7% (39/827) were unmatched. A detailed breakdown of the semantically similar terms identified by the validators is shown in Table 3. The agreement between the 2 validators was strong, with a Cohen κ of 0.88 (95% CI 0.80‐0.95) [43]. All automatically mapped terms were accepted by both validators.

We identified 42 terms where discrepancies arose between the validators, which were resolved through consensus discussions. One source term (ie, “endotracheal intubation”) was deemed irrelevant as it represents a clinical procedure rather than an ADE symptom or diagnosis and was therefore removed. A key point during the discussions for the remaining terms was the user-friendliness of the mapped terms. Validators noted that in some cases, semantically similar terms were preferred over semantically equivalent ones for ease of use. For instance, the term “sunburn” had an equivalent match with |403194002| “Solar erythema (disorder),” but the validators flagged this term as non–user-friendly due to lack of clinical use, recommending that “sunburn” to be added as a synonym in the Canada English language reference set. Table 4 shows a list of terms, like these, that were flagged by the map validators due to clinical usability issues.

The validated map included 826 source terms, with 98.4% (813/826) mapped to a target SNOMED CT concept, and 1.6% (13/826) remaining unmapped. Of the mapped terms, 95.7% (778/813) were semantically equivalent, 2.5% (20/813) were broader, 1.0% (8/813) were narrower, and 0.9% (7/813) were inexact matches.

**Table 3.** Breakdown of semantically similar terms suggested by validators.

Category	Validator 1^a^	Validator 2^b^
Semantically broader than the source term	12	13
Semantically narrower than the source term	6	8
Semantically inexact to the source term	7	9
Total	25	30

a Validator 1 suggested 25 target terms to describe 25 source terms without a semantically equivalent match, resulting in an average of 1.0 target term per source term.

b Validator 2 suggested 30 target terms to describe 25 source terms without a semantically equivalent match, resulting in an average of 1.2 target terms per source term.

**Table 4.** A list of mapped terms flagged by validators due to clinical usability issues.

Source code	Source display	Target code	Target display	Relationship type	Summary of validator comments
10029147	Nephrogenic diabetes insipidus	111395007	Arginine vasopressin resistance (disorder)	Equivalent	Non–user-friendly. Recommend adding this as a synonym in the Canada English language reference set.
10042496	Sunburn	403194002	Solar erythema (disorder)	Equivalent	Non–user-friendly. Recommend adding “sunburn” as a synonym in the Canada English language reference set.
10066371	Tendon pain	21545007	Tenalgia (finding)	Broader	Non–user-friendly. Recommend adding “Tendon pain” as a synonym in the Canada English language reference set.
10073183	Hyponatremic seizure	230358007	Hyponatremic encephalopathy (disorder)	Equivalent	Non–user-friendly. Recommend adding “Hyponatremic seizure”. as a synonym in the Canada English language reference set.
10080061	Euglycemic diabetic ketoacidosis	420422005	Ketoacidosis due to diabetes mellitus (disorder)	Broader	Mapped to a broader term, but recommend creating a subtype of this for “Euglycemic diabetic ketoacidosis.”

### Quality Review and Create Final Value Set

The quality review team flagged 6 terms for further discussion due to potential safety risks or clinical relevance issues (Table 5). The project team reached a consensus on all flagged terms. One term was removed for being irrelevant to ADE documentation, while 5 were left unmapped, with recommendations for requesting new SNOMED CT codes to be added to the SNOMED CT Canadian edition. Similar to the validators’ feedback, 2 quality reviewers flagged the term “solar erythema (disorder)” for lacking clinical usability, with recommendations that the term “Sunburn” be used as the preferred term in the ADE clinical context.

The quality reviewers agreed that the final map included most major ADE symptoms and diagnosis terms commonly encountered in clinical practice, and no additional source terms were suggested (Multimedia Appendix 2). Based on this feedback, we created the ADE value set, consisting of 813 SNOMED CT concepts (Multimedia Appendix 3). There are 13 source terms awaiting new SNOMED CT concepts, which could be added to the value set in a later phase (Table 6).

**Table 5.** A list of terms flagged by quality reviewers for potential safety risks and issues related to clinical relevance.

Source code	Source display	Target code	Target display	Relationship type	Summary of quality reviewer comments
10005140	Bleeding time prolonged	165563002	Coagulation/bleeding tests abnormal (finding)	Broader	“Prolonged bleeding time” is more accurate. The term “bleeding time” is commonly used by both clinicians and in lay language, making it a reasonable match.
10028128	Mucositis management	N/A^a^	N/A	Unmapped	The “management” term does not seem to fit under ADE^b^ diagnosis and symptoms. If included, it should be simplified to “mucositis.” They also suggest using consistent terminology with other coding, and “inflammatory disease of mucous membrane” could be an appropriate replacement for “mucositis.”
10071048	Seizure-like phenomena	1208961006	Nonmotor epileptic seizure (finding)	Inexact	Both terms are not ideal. “Seizure-like phenomena” is not equivalent to “nonmotor epileptic seizure,” with one noting that it is too vague and the other highlighting its potential relevance as a distinct term.
10080061	Euglycemic diabetic ketoacidosis	420422005	Ketoacidosis due to diabetes mellitus (disorder)	Broader	Both “Ketoacidosis due to diabetes mellitus” and “Euglycemic DKA^c^” should be available.

a N/A: not applicable.

b ADE: adverse drug event.

c DKA: diabetic ketoacidosis.

**Table 6.** A list of source terms pending new target SNOMED CT codes.

Source code	Source display
10005140	Bleeding time prolonged
10028299	Muscle discomfort
10031118	Oropharyngeal swelling
10048868	Parkinsonian crisis
10061132	Drug level above therapeutic
10061642	Antibiotic level above therapeutic
10062015	Immunosuppressant drug level increased
10067360	Tongue necrosis
10067969	Cholestatic liver Injury
10079741	Sedation complication
10000489	Acidosis hyperchloremic
10071048	Seizure-like phenomena
10080061	Euglycemic diabetic ketoacidosis

## Discussion

### Principal Results

This project aimed to develop a standardized SNOMED CT–based value set for the documentation of ADE symptoms and diagnoses, addressing a critical gap in the integration of ADE reporting into EMRs. Our findings demonstrate the feasibility of creating a targeted yet comprehensive value set, which can enhance the consistency, accuracy, and interoperability of ADE documentation across health care systems.

The mapping of 826 unique ADE-related terms to SNOMED CT concepts was largely successful, with 98.3% of terms being mapped in the final value set. This high level of success reflects the comprehensiveness of the SNOMED CT framework and the effectiveness of our systematic mapping process. Strong agreement between mappers and between validators supports the reliability of the mapping. Moreover, the iterative process of consensus building for resolving discrepancies between mappers and validators underscores the importance of expert input in ensuring the clinical appropriateness of mapped terms.

A major challenge encountered was ensuring semantic equivalence while maintaining clinical usability. While semantic equivalence is critical for accurate mapping, as SNOMED CT serves as the reference terminology, achieving clinical usability requires careful management of interface terminology. This can involve using SNOMED CT’s dialect-specific language reference sets, also known as the preferred term (eg, en-CA or specialized ADE-context dialects), or establishing one-to-one association between value set entries and interface terms.

In SNOMED CT, the FSN provides an unambiguous and stable description of a concept but is not always clinician-friendly or commonly used [44]. In contrast, the preferred term is the most clinically appropriate and widely used synonym in a given dialect [45]. Therefore, interface terminology should default to the concept’s preferred synonym in the give dialect, not the FSN. For example, the source term “gout” maps to the equivalent concept 90560007, with an FSN of inflammatory disorder due to increased blood urate level—a term less familiar in practice. The preferred term in the Canada English reference set, gout, is more user-friendly and should be displayed in clinical interfaces. While display codes can help improve terms usability, clinical reviewers noted that this work-around solution does not resolve the underlying issue of terms lacking clinical usability.

Validators and quality reviewers also flagged terms where the preferred terms in the Canada English language reference set, while semantically equivalent to SNOMED CT concepts, were still not intuitive for clinical documentation. For example, the source term “sunburn” has a preferred term as “solar erythema.” Reviewers noted that they had never before heard of or used this term clinically, ever. This highlights the importance of considering both semantic equivalency and clinical usability when developing standardized terminologies for real-world application and minimize barriers for users [35].

Future research should prioritize understanding the clinical usability of standardized terminologies and developing interface terminology that users can understand and apply. While SNOMED CT provides a robust framework for clinical documentation, more work is needed to ensure that mapped terms are intuitive and align with the language clinicians commonly use in practice. It is critical that an effective approach is adopted to supporting interface terms that will enable value sets to be clinically useful. Without suitable interface terms, clinicians may struggle to recognize or apply relevant concepts within clinical systems, limiting the practical use of mapped value sets.

### Clinical Implications of the ADE Value Set

The adoption of this value set for ADE documentation holds significant implications for clinical practice. By standardizing the terminology used in EMRs, health care providers can improve the accuracy of ADE reporting, leading to better communication across care teams and potentially reducing the risk of medication-related harm. The use of standardized terminology can facilitate data sharing across health care institutions and enhance pharmacovigilance efforts at the population level, improving patient safety, if the code set can be used and understood by clinicians. Moreover, the standardized terminology provided by this value set could enhance the performance of text-mining algorithms designed to identify ADEs in EMRs [46]. However, the success of this value set depends not only on its development but also on its integration into EMR systems and clinical workflow. A key challenge lies in the willingness of EMR vendors to incorporate value sets, such as importing standardized value sets external to the Cerner system.

To ensure the successful adoption of the SNOMED CT–based ADE value set, provincial or federal governments may need to engage with EMR vendors. Governments could explore ways to encourage or mandate vendors to engage with clinical users to support the integration of locally relevant value sets, either through regulatory frameworks or through financial incentives. Without such support, the clinical benefits of standardized ADE documentation may be difficult to achieve.

In addition, the success of the value set relies on its integration into clinical workflows. Ensuring that the terminology is not only accurate but also practical and intuitive for day-to-day use will be critical. Ongoing collaboration between technical experts, clinical users, and policy makers will be essential to make certain that the value aligns with current clinical documentation practices and meets the needs of health care providers. Additionally, it is crucial to regularly update the terms to reflect the modern language and evolving practices in health care. As medical terminology changes and evolves, ensuring that value sets remain current will maintain their relevance and improve their usability in real-world clinical settings. By addressing these challenges, the value set has the potential to significantly improve the quality and consistency of ADE reporting, ultimately enhancing patient care.

Ineffective communication of ADE information across health care settings can lead to repeat ADEs, partly because different EMR systems use varied terminology standards. Recognized as a Canadian national standard and widely adopted in clinical areas including e-prescribing [47], our SNOMED CT value set adds values by standardizing ADE documentation in EMRs and enabling seamless ADE data exchange between hospital and community settings. This will allow clinicians to have timely access to patient’s ADE data at the point of care, preventing unintentional represcription or redispensation of culprit drugs, thereby reducing the incidence of repeat ADEs.

### Limitations

It is important to note that our study extracted source terms from a single ADE reporting system—ActionADE—which captures ADEs from patients presenting predominantly in emergency departments across 9 hospitals within a specific geographic region during a specific time period. As a result, the symptoms and diagnoses captured in this context may not fully represent ADEs occurring in other health care settings, such as inpatient wards, community care, or long-term care facilities, where different patient populations and clinical presentations are more common. For example, ADEs in long-term care settings may involve different drug classes and clinical manifestations than those seen in emergency departments. Similarly, pediatric ADEs were underrepresented in our sample, as none of the included hospitals were dedicated pediatric centers. Finally, as new drugs are developed and use of existing drugs is expanded to new clinical indications, new ADEs may emerge over time. Future work will need to expand the scope of this project by exploring and refining the value set to ensure its applicability across a diverse range of clinical environments, patient populations, and over time.

We acknowledge that using MedDRA’s broad, high-level terms as our source may limit the potential granularity of our resulting SNOMED CT value set. While our approach may not have enhanced granularity of the resulting value set, the benefits of SNOMED CT’s superior semantic framework, logical definition, and hierarchical structure, along with its increasing adoption as a Canadian national standard, justify its use in creating an initial ADE value set. Future research should explore the impact of using more granular source terminologies on the resulting SNOMED CT value set.

Another limitation is the potential variability in how individual reviewers interpreted ADE-related terms, which may have been influenced by their clinical background, experience, or familiarity with SNOMED CT concepts. While mapping should be grounded in semantic equivalence to ensure technical accuracy, variability may have occurred when terms had multiple valid mappings depending on the clinical context. These discrepancies highlight the need for better education on the importance of mapping based on meaning and semantics only, and tooling needs to provide better support for the incorporation of interface terms into terminology service using additional synonyms and a context-based language reference set.

Additionally, due to vendor limitations, we were unable to test the value set in the context where it will be used to ensure that it meets the needs of relevant stakeholders. Further studies are required to evaluate the impact of adopting standardized ADE value sets on clinical outcomes. Research examining how the integration of a SNOMED CT–based value set in EMRs affects the quality of ADE reporting, patient safety, and pharmacovigilance on a larger scale will be crucial to demonstrate the real-world benefits of this approach.

### Conclusions

The development of a SNOMED CT–based value set for the documentation of ADE symptoms and diagnoses represents an important step toward improving the standardization of clinical documentation and enhancing patient safety. While the mapping process was largely successful, ongoing efforts to refine and expand the value set, improve user-friendliness, and evaluate its impact on clinical practice will be critical for ensuring its long-term adoption and use. By integrating this value set into clinical workflows and continuing to address gaps in terminology, health care systems can enhance the quality and interoperability of ADE documentation, contributing to better patient care.

## Supplementary material

10.2196/70167 Multimedia Appendix 1 Screenshots of the Snap2SNOMED platform.

10.2196/70167 Multimedia Appendix 2 MedDRA-to-SNOMED CT map for adverse drug event symptoms and diagnoses.

10.2196/70167 Multimedia Appendix 3 SNOMED CT–based value set for adverse drug event symptoms and diagnoses.

## Abbreviations

ADE: adverse drug event
EMR: electronic medical record
FSN: fully specified name
ICD-10: International Classification of Diseases, Tenth Revision
MedDRA: Medical Dictionary for Regulatory Activities
SNOMED CT: SNOMED Clinical Terms
