# Supplementary material for: Developing a SNOMED CT–Based Value Set to Document Symptoms and Diagnoses for Adverse Drug Events: Mixed Methods Study
Source: JMIR Med Inform. 2025 Jul 8;13:e70167. doi: 10.2196/70167 (PMC12262102; doi:10.2196/70167)
Supplement: Multimedia Appendix 1 [file medinform-v13-e70167-s001.pdf]

Multimedia Appendix 1. Screenshots of the Snap2Snomed platform

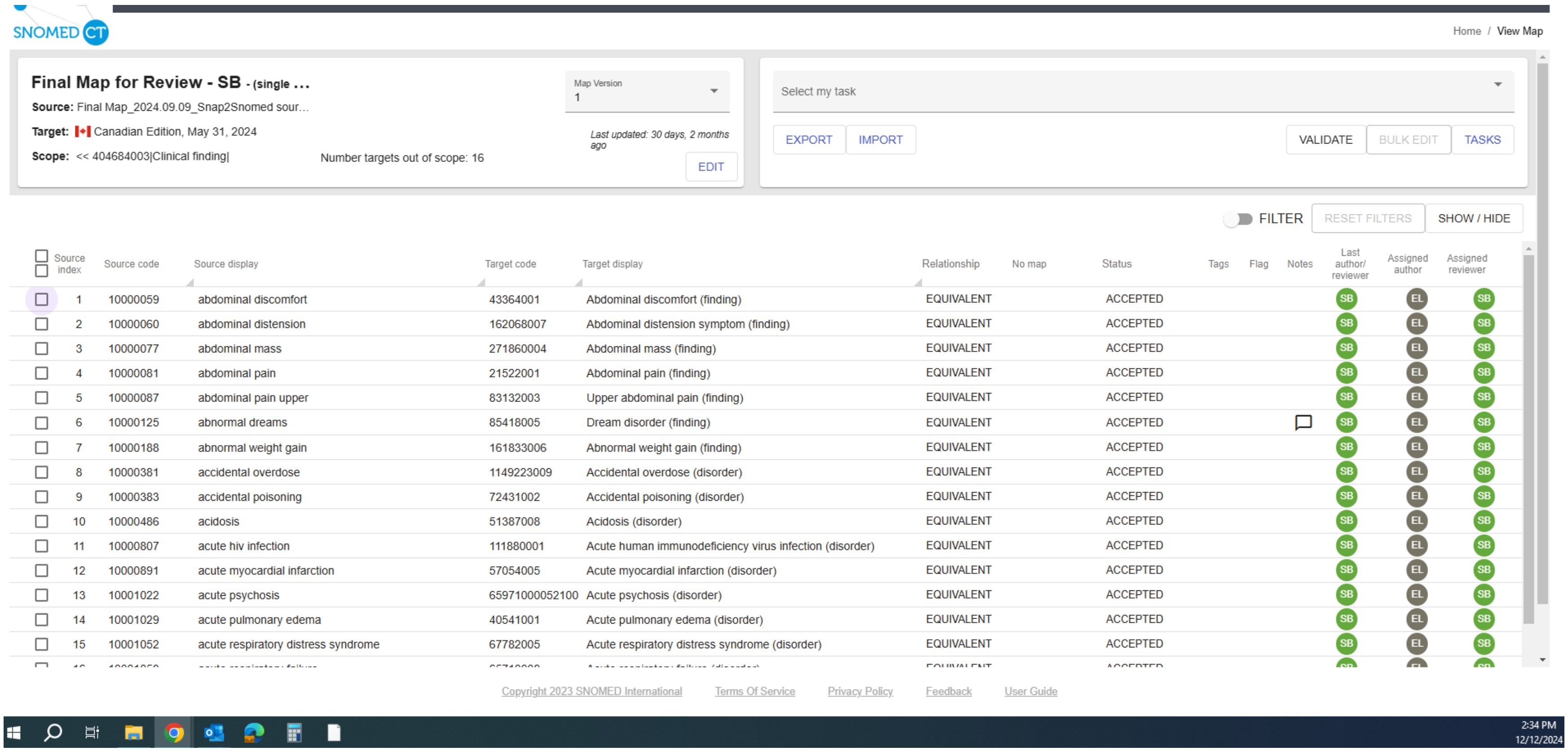

Source index	Source code	Source display	Target code	Target display	Relationship	No map	Status	Tags	Flag	Notes	Last author/ reviewer	Assigned author	Assigned reviewer
1	10000059	abdominal discomfort	43364001	Abdominal discomfort (finding)	EQUIVALENT		ACCEPTED				SB	EL	SB
2	10000060	abdominal distension	162068007	Abdominal distension symptom (finding)	EQUIVALENT		ACCEPTED				SB	EL	SB
3	10000077	abdominal mass	271860004	Abdominal mass (finding)	EQUIVALENT		ACCEPTED				SB	EL	SB
4	10000081	abdominal pain	21522001	Abdominal pain (finding)	EQUIVALENT		ACCEPTED				SB	EL	SB
5	10000087	abdominal pain upper	83132003	Upper abdominal pain (finding)	EQUIVALENT		ACCEPTED				SB	EL	SB
6	10000125	abnormal dreams	85418005	Dream disorder (finding)	EQUIVALENT		ACCEPTED				SB	EL	SB
7	10000188	abnormal weight gain	161833006	Abnormal weight gain (finding)	EQUIVALENT		ACCEPTED				SB	EL	SB
8	10000381	accidental overdose	1149223009	Accidental overdose (disorder)	EQUIVALENT		ACCEPTED				SB	EL	SB
9	10000383	accidental poisoning	72431002	Accidental poisoning (disorder)	EQUIVALENT		ACCEPTED				SB	EL	SB
10	10000486	acidosis	51387008	Acidosis (disorder)	EQUIVALENT		ACCEPTED				SB	EL	SB
11	10000807	acute hiv infection	111880001	Acute human immunodeficiency virus infection (disorder)	EQUIVALENT		ACCEPTED				SB	EL	SB
12	10000891	acute myocardial infarction	57054005	Acute myocardial infarction (disorder)	EQUIVALENT		ACCEPTED				SB	EL	SB
13	10001022	acute psychosis	65971000052100	Acute psychosis (disorder)	EQUIVALENT		ACCEPTED				SB	EL	SB
14	10001029	acute pulmonary edema	40541001	Acute pulmonary edema (disorder)	EQUIVALENT		ACCEPTED				SB	EL	SB
15	10001052	acute respiratory distress syndrome	67782005	Acute respiratory distress syndrome (disorder)	EQUIVALENT		ACCEPTED				SB	EL	SB

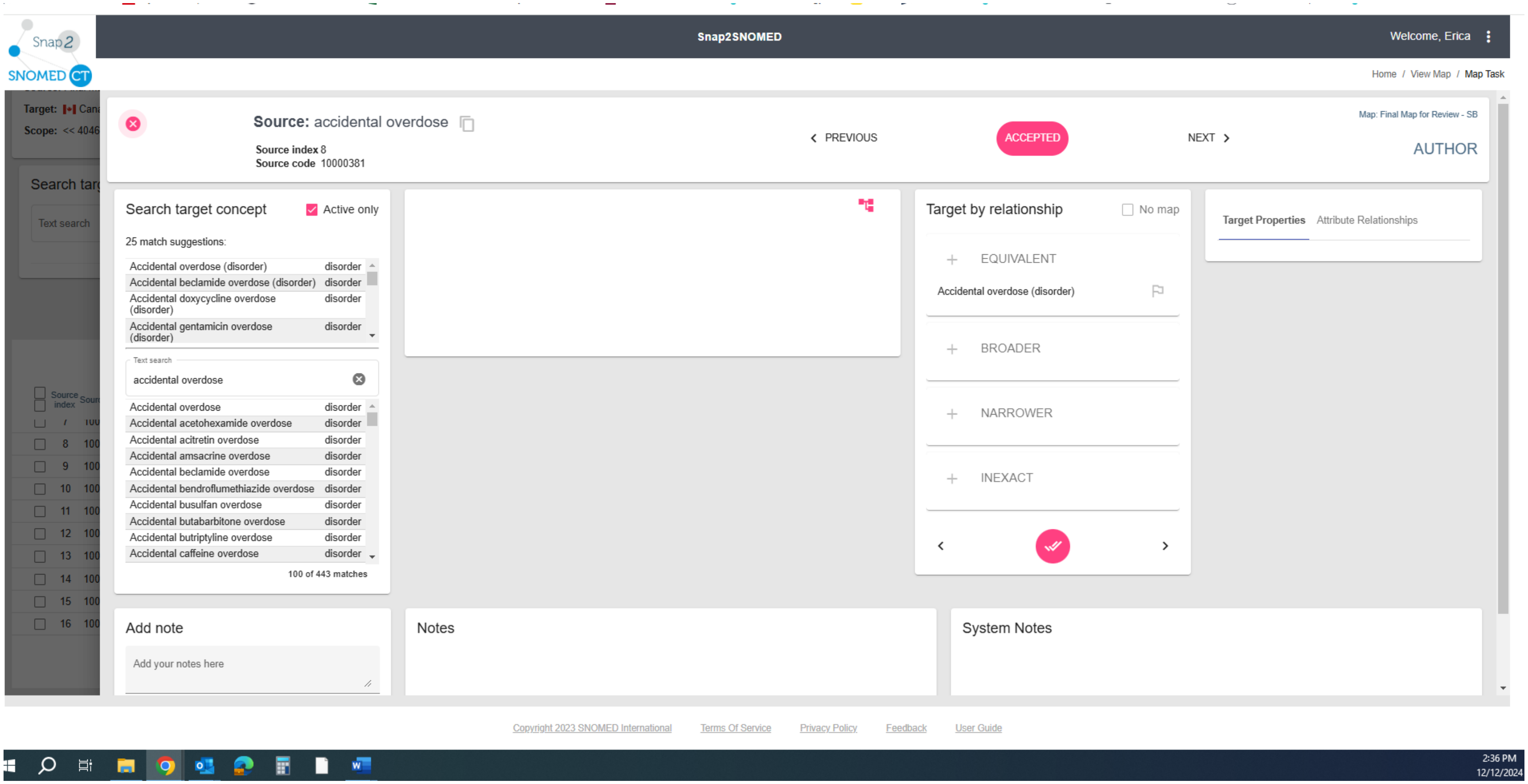
Snap2
SNOMED CT
Snap2SNOMED
Welcome, Erica
Home / View Map / Map Task
Target:
Scope: << 4046
Search targ
Text search
Source: accidental overdose
Source index 8
Source code 10000381
PREVIOUS
ACCEPTED
NEXT
Map: Final Map for Review - SB
AUTHOR
Search target concept
Active only
25 match suggestions:
Accidental overdose (disorder) disorder
Accidental beclamide overdose (disorder) disorder
Accidental doxycycline overdose (disorder) disorder
Accidental gentamicin overdose (disorder) disorder
Text search
accidental overdose
Accidental overdose disorder
Accidental acetohexamide overdose disorder
Accidental acitretin overdose disorder
Accidental amsacrine overdose disorder
Accidental beclamide overdose disorder
Accidental bendroflumethiazide overdose disorder
Accidental busulfan overdose disorder
Accidental butabarbitone overdose disorder
Accidental butriptyline overdose disorder
Accidental caffeine overdose disorder
100 of 443 matches
Target by relationship
No map
EQUIVALENT
Accidental overdose (disorder)
BROADER
NARROWER
INEXACT
Target Properties
Attribute Relationships
Add note
Add your notes here
Notes
System Notes
Copyright 2023 SNOMED International
Terms Of Service
Privacy Policy
Feedback
User Guide
2:36 PM
12/12/2024
